# Fallopian Canal Meningocele Causing Cerebrospinal Fluid Rhinorrhoea

**DOI:** 10.1007/s12070-023-03963-3

**Published:** 2023-06-21

**Authors:** Miguel Saro-Buendía, Lidia Torres-García, Santiago Almanzo, Daniel Felipe Mora Aristizabal, Carlos de Paula Vernetta, Miguel Armengot Carceller

**Affiliations:** 1 Servicio de Otorrinolaringología, Hospital Universitario y Politécnico La Fe, Avinguda Fernando Abril Martorell 106, València, 46026 España; 2 Departament de Cirugia, Facultat de Medicina i Odontología, Universitat de València, València, España; 3 Servicio de Radiología, Hospital Universitario y Politécnico La Fe, València, España

**Keywords:** Fallopian canal meningocele, Cerebrospinal fluid rhinorrhoea, Intratemporal facial nerve anomalies, Cerebrospinal fluid leaks

## Abstract

Fallopian canal meningocele is an extremely rare cause of cerebrospinal fluid rhinorrhoea. Also, due to complex anatomical relations and a lack of experience, its management remains a challenge. Here we report a case focusing on its clinical course, radiological features, and management.

## Introduction

Fallopian canal meningocele (FCM) is a rare cause of cerebrospinal fluid (CSF) leak. Only 17 cases have been reported to date, so its incidence is unknown [1–8]. Pathophysiology is not clear and there are several hypotheses available [1]. Management of FCM is complex due to the intimate anatomical relationship between meningocele and the intratemporal facial nerve. Moreover, still there are no guidelines for the management of this condition [1].

## Clinical Case

A 6-year-old female visited our centre, she had history of idiopathic intracranial hypertension and recurrent meningitis. Moreover, she had history of a left medial temporal lobe encephalocele managed surgically by transsphenoidal approach in another centre.

**Fig. 1:**
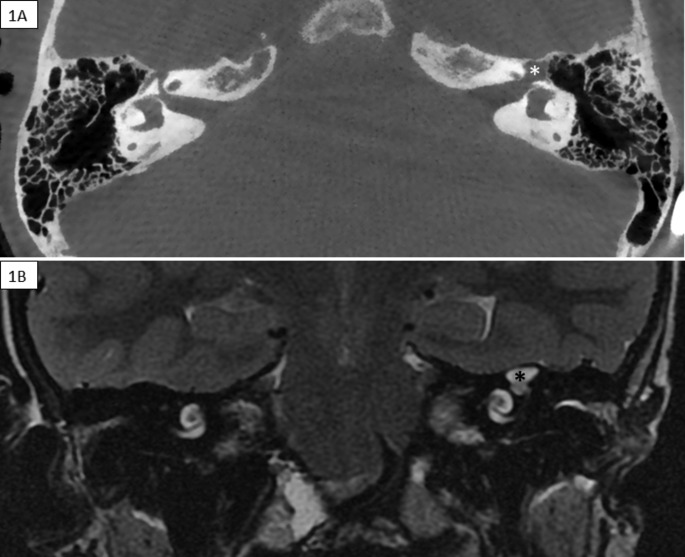
**A** (Cone Beam CT) and **B** (MRI, T2 sequence): Fluid ectasia in the left geniculate ganglion (*) in continuity with the tympanic and labyrinthine segments of the facial nerve. There are no bony defects communicating the ipsilateral temporal fossa

**Fig. 2:**
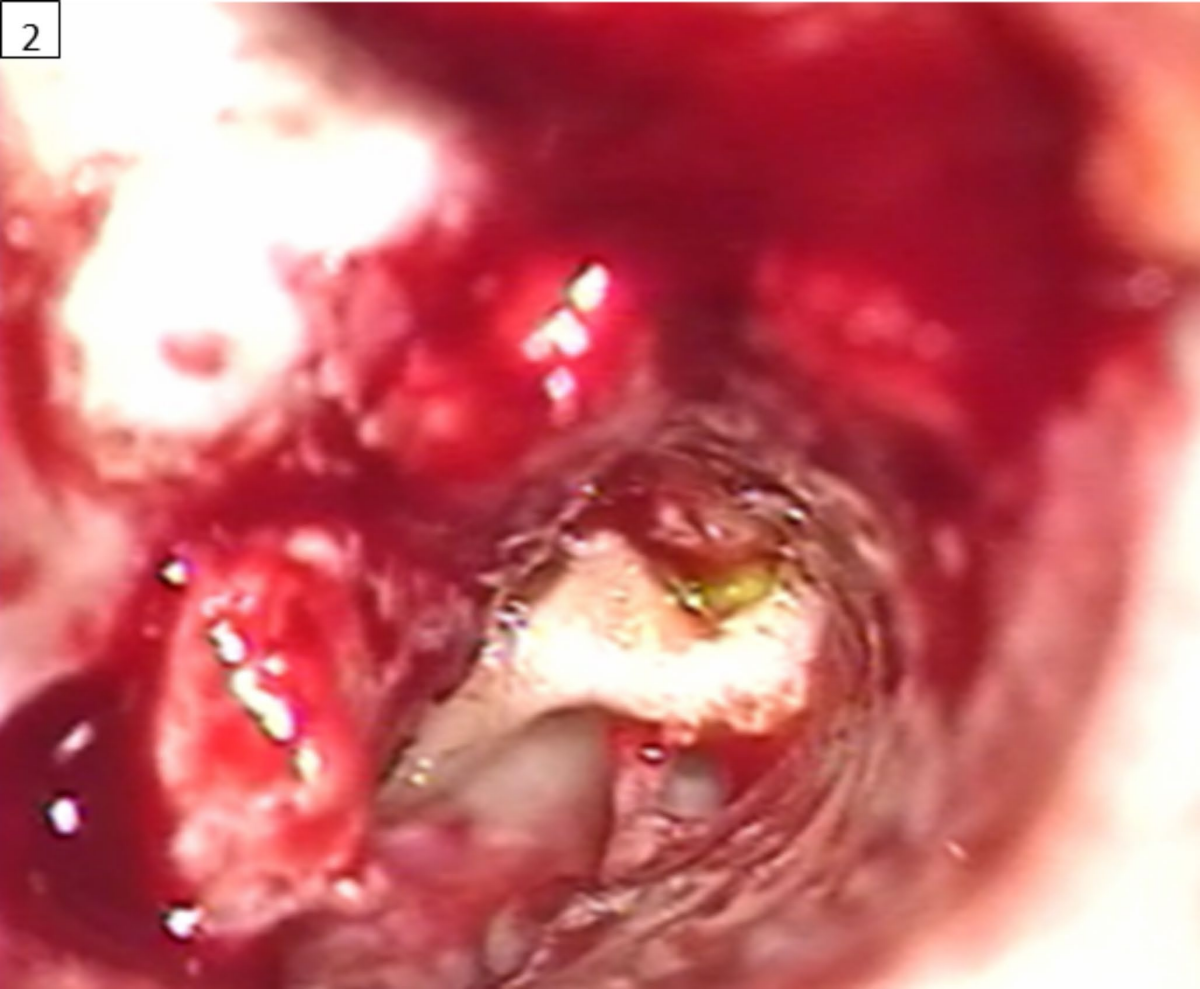
Left exploratory tympanotomy after the injection of intrathecal fluorescein. Fluorescein is observed sliding over the malleus head. Then, the presence of a CSF leak is confirmed

She presented to our centre with suspicion of intermittent CSF rhinorrhoea. A CSF leak was detected at the base of both pterygoid processes. Neurosurgery tried to seal these leaks surgically. However, rhinorrhoea persisted and a right- sided intermittent facial palsy debuted. Then, a ventriculoperitoneal shunt was performed ceasing rhinorrhoea complaints. Nonetheless, two months later the leak was evidenced again, and we decided to explore the nasopharynx surgically using intrathecal fluorescein. The substance was observed in the nasopharyngeal mucosa, especially around the left eustachian tube opening. Cone beam computed tomography (CT) and magnetic resonance were performed to explore the left temporal bone and middle cranial fossa and findings were compatible with FCM (Fig. 1 A and 1.B). Then, a left exploratory tympanotomy, under the use of intrathecal fluorescein, was performed. CSF leak was evidenced sliding over the malleus head (Fig. 2). An atticotomy was performed and the origin of the CSF leak was observed in geniculate ganglion area close to the supratubaric fossa. The leak was repaired with TachoSil®, conchal cartilage and temporal fascia. Four months after surgery, she was admitted again due to suspicion of CSF otorrhea but beta-2-transferrin tests were negative. Then, were performed a radionuclide cisternography with intrathecal 111In-DTPA and a SPECT/CT, both with negative results for CSF leak. Now, she is asymptomatic and has regular ambulatory follow up.

## Discussion

FCM has been described as the underlying cause of a CSF leak in 17 cases, the first of them was reported in 1967 [1–8]. There is no apparent sex predilection and cases have been described in patients aged from 2 to 64 years old [1]. Symptoms are usually unilateral and include ear fullness and conductive hypoacusis. In some cases, like ours, CSF leaves the middle ear through the eustachian tube conditioning a CSF rhinorrhoea. More than half of the patients had history of recurrent meningitis. However, the are no reports of previous facial palsy.

Geniculate fossa is the dehiscence location in our patient and in more than 70% of the cases reported [1]. Theoretically, CT shows a wide geniculate fossa in continuity with a short and wide labyrinthine segment. On the other hand, MRI shows an hyperintensity on T2W sequencies representing an arachnoid herniation filled by CSF [3]. A hypothesis is that a wide labyrinthine segment might condition the lateralization of the subarachnoid space towards the fallopian canal. Then, pressure and CSF pulsations at this area might cause bony erosion and dehiscence conditioning a lateral leptomeningeal herniation [2, 5, 7, 9].

The management is challenging compared to usual CSF leak locations (tegmen tympani and mastoid region). First, in case of FCM, the CSF leak has its origin on the posterior fossa and these leaks have higher CSF flow than middle fossa leaks. Moreover, the surgical management of FCM requires the occlusion of the fallopian canal avoiding damage to the facial nerve [1]. Concretely, the surgical approach may be trans mastoid (as our case), middle fossa craniectomy or combined. Generally, a middle fossa craniectomy or a combined approach seem to be more appropriate for a geniculate ganglion dehiscence. However, the fallopian canal dehiscence is controlled by a trans mastoid approach. Temporal fascia or muscle are used to seal the CSF leak [1]. Postoperatively, 20% of the patients presented facial palsy but our patient did not. Also, 27% of the patients presented again a CSF leak after surgery. Our patient was hospitalized due to CSF leak suspicion. However, beta-2-transferrin test, radionuclide cisternography with intrathecal 111In-DTPA and SPECT/CT were all negative [1]. As our patient, cases with refractory CSF leaks seem to have history of idiopathic intracranial hypertension. Episodes of CSF leak require placement of lumbar drainage and observation, surgical sealing of the CSF leak or a ventriculoperitoneal shunt. Subtotal petrosectomy and external ear canal closure might be appropriate for patients who do not accept the possibility of facial nerve injury or persistent CSF otorrhoea [1, 6].

## Conclusions

To summarize, FCM is a rare cause of CSF leaks and only 17 cases have been reported to date. Surgical management is complex and there is a high risk of postoperative facial nerve palsy and/ or persistence of the CSF leak. Then, to report experiences is essential to precisely assess risks and optimize the management of future cases.

## Abbreviations

FCM: Fallopian canal meningocele
CSF: cerebrospinal fluid
CT: computed tomography
